# miR-125 in Breast Cancer Etiopathogenesis: An Emerging Role as a Biomarker in Differential Diagnosis, Regenerative Medicine, and the Challenges of Personalized Medicine

**DOI:** 10.3390/ncrna10020016

**Published:** 2024-02-21

**Authors:** Roberto Piergentili, Enrico Marinelli, Gaspare Cucinella, Alessandra Lopez, Gabriele Napoletano, Giuseppe Gullo, Simona Zaami

**Affiliations:** 1 Institute of Molecular Biology and Pathology, Italian National Research Council (CNR-IBPM), 00185 Rome, Italy; roberto.piergentili@cnr.it; 2 Department of Medico-Surgical Sciences and Biotechnologies, “Sapienza” University of Rome, 04100 Latina, Italy; enrico.marinelli@uniroma1.it; 3 Department of Obstetrics and Gynecology, Villa Sofia Cervello Hospital, University of Palermo, 90146 Palermo, Italy; gaspare.cucinella@unipa.it (G.C.); alessandralopez91@gmail.com (A.L.); gullogiuseppe@libero.it (G.G.); 4 Department of Anatomical, Histological, Forensic and Orthopedic Sciences, Section of Forensic Medicine, “Sapienza” University of Rome, 00161 Rome, Italy; gabriele.napoletano@uniroma1.it

**Keywords:** breast cancer (BC), MicroRNA (miR), non-coding RNA, competing endogenous RNA (ceRNA), personalized medicine, ethical and legal challenges

## Abstract

Breast Cancer (BC) is one of the most common cancer types worldwide, and it is characterized by a complex etiopathogenesis, resulting in an equally complex classification of subtypes. MicroRNA (miRNA or miR) are small non-coding RNA molecules that have an essential role in gene expression and are significantly linked to tumor development and angiogenesis in different types of cancer. Recently, complex interactions among coding and non-coding RNA have been elucidated, further shedding light on the complexity of the roles these molecules fulfill in cancer formation. In this context, knowledge about the role of miR in BC has significantly improved, highlighting the deregulation of these molecules as additional factors influencing BC occurrence, development and classification. A considerable number of papers has been published over the past few years regarding the role of miR-125 in human pathology in general and in several types of cancer formation in particular. Interestingly, miR-125 family members have been recently linked to BC formation as well, and complex interactions (competing endogenous RNA networks, or ceRNET) between this molecule and target mRNA have been described. In this review, we summarize the state-of-the-art about research on this topic.

## 1. Introduction

Breast Cancer (BC) is the most commonly diagnosed cancer type, accounting for one in seven cancer diagnoses [1]. Available data show that incidence and mortality in developed countries have been declining over time, while they have increased in low-income countries [2]. In 2020, there were about 2.3 million new cases of BC globally and about 685,000 deaths from this disease, with large geographical variations reported in individual countries and world regions. BC incidence rates are highest in developed countries, whereas developing countries have a disproportionately high share of BC deaths [3]. In the United States, BC alone is expected to account for more than 30% of all new cancers in women [1]. The 2018 GLOBOCAN (Global Cancer Data) shows that age-standardized incidence rates (ASIR) of BC are strongly and positively associated with the Human Development Index (HDI) [4]. The HDI is a statistical composite index developed by the United Nations Development Programme’s (UNDP) Human Development Report Office to measure life expectancy at birth, education, and national *per capita* incomes. The above-mentioned study focused on BC expanding and completing the HDI data by considering additional indexes. Data reported by Sharma indicate that the high incidence and high survival rates in developed countries probably reflect BC early detection, likely due to better cancer infrastructures available (e.g., hospitals), systematic screening programs (e.g., breast mammograms), and more efficient BC treatment in these countries, which low HDI countries do not possess.

## 2. Clinical Features of BC

### 2.1. Main Risk Factors of BC

A variety of risk factors for BC have been well-established by epidemiologic studies and include ethnicity as well as behavioral variables, such as sedentary lifestyle or increased alcohol consumption. Overdrinking can, in fact, elevate estrogen-related hormone levels in the blood and trigger estrogen receptor pathways [5]. In addition, endogenous and exogenous estrogens are associated with an increased risk of BC. Endogenous estrogen is usually produced by the ovary in premenopausal women, and ovariectomy can reduce the risk of BC. The main sources of exogenous estrogen are oral contraceptives and hormone replacement therapy (HRT) [6]. Certain female reproductive factors, such as younger age at menarche, low parity, late menopause, and older age at first full-term pregnancy, may influence BC risk through long-term effects on sex hormone levels or by other biological mechanisms, although recent studies suggest that triple negative BC may have a distinct etiology [7]. Over the past decades, the incidence of pregnancy-associated BC (PABC) has been on the rise as well [8]. Lastly, having extremely dense breast tissue is also significantly associated with increased BC risk compared to having scattered dense breast tissue [9].

Nearly one-fourth of all BC cases are related to family medical history: women whose mothers or sisters have BC are more prone to developing this disease [10], indicating a strong genetic basis in its etiology. Indeed, the inherited susceptibility to BC is attributed to mutations in BC-related genes such as *BRCA1* and *BRCA2* [11]. Additional genes that, during the years, have been implicated in BC pathogenesis include *PTEN* [12], *TP53* [13], *CDH1* [14], *STK11* [15], *CHEK2* [16], *PALB2* [17], *ATM* [18], *RAD51C* [19], *RAD51D* [20], *BARD1* [21], *NF1* [22], *BRIP1* [23], *CASP8*, *CTLA4*, *NBN,* and, possibly, *CYP19A1*, *TERT* and *XRCC3* [24]. A summary of the most frequently mutated genes in BC, their function and their relation to BC is summarized in Table 1.

### 2.2. BC Characterization and Patient Management

BC is commonly diagnosed via ultrasonography [29,30]. Mammography screening for malignancy is commonly used as well to detect the disease [31], while breast magnetic resonance imaging (MRI) is used in conjunction with mammography as a support tool [32]. In this case, MRI can be helpful in deciding whether to have a breast-conserving mastectomy or surgery [33,34]. A biopsy is performed when mammograms, other imaging tests, or a physical exam show a breast change that may be identified as a possible cancerous mass. Computerized tomography (CT) scans, MRIs, ultrasound, and positron emission tomography (PET) scans may also provide information as to cancer extension and position. Laboratory tests of cancer cells (from biopsy or surgery) and blood tests can also be used to help stage some types of cancer [35]. Recently developed techniques for BC diagnosis include Digital Breast Tomosynthesis (DBT), which is a subset of the mammography procedure [36], and the Contrast-enhanced digital mammography (CEDM), which represents the angiogenic pattern of the masses and allows depicting the anatomical information of the tissue [37]. Each of these methods has different subdivisions and their advantages and disadvantages have been discussed in the literature [38,39,40]. Although there are ways to improve these methods, it should be kept in mind that simultaneously combining multiple imaging techniques would significantly improve BC early detection [41].

The stage of a cancer is helpful in assessing its extension and spreading. The tumor-node-metastasis (TNM) staging system (Table 2) is currently the most widespread method to stage BC. However, staging for BC can be very complex; many different factors should be accounted for before the cancer stage can be confirmed to outline the most suitable therapeutic approach. The TNMEIO system was suggested by the European Institute of Oncology (EIO) to include tumor characteristics affecting treatment decisions in the TNM system [42].

Perou and Sorlie proposed the “Molecular Classification” terminology in BC for the first time in 2000 with a comprehensive study showing the differences in gene expression of different BC specimens. In this study, breast tissue samples were divided into different sub-groups according to variable gene expression, i.e., ER+/luminal-like, basal-like, Erb-B2+ and normal breast [43,44].

The complexity of BC staging is also reflected in the different histological classifications used to identify its various subtypes. This has led to defining different BC types, such as ductal carcinoma (in situ or invasive), medullary carcinoma, lobular carcinoma (in situ or infiltrating), tubular and mucinous carcinoma. Expression analysis of additional molecular markers such as the estrogen receptor (ER), progesterone receptor (PR) and HER2/neu proteins can provide further, highly valuable knowledge for the oncologist. Once the status of these proteins is known, the prognosis can be reasonably predicted, and more appropriate therapies may be chosen for treatment [45]. Recently, the eighth edition of the American Joint Commission of Cancer (AJCC) staging system for BC approved major changes in the classification system, adopting an anatomy-based and histology-based subdivision built on the original TNM staging system and adding various biomarkers to refine the prognostic information for better selection of therapy with improved outcome [46]. This finer classification should contribute to better characterizing the molecular and anatomical features of each patient and delineating a therapeutic pathway accordingly. Thus, it is crucial for oncologists to have the most comprehensive description of BC, both from a histological and a molecular standpoint.

BC therapy involves a multidisciplinary approach relying on surgery, radiotherapy, chemotherapy, hormone therapy, immunotherapy, neoadjuvant and adjuvant therapy. Effective BC therapy must aim for maximum therapeutic efficacy [47]. There is increasing recognition that the care of a BC patient depends on highly individualized clinical features, including the stage at presentation, the biological subset of BC, the genetic factors that may underlie BC risk, the genomic signatures that advise treatment recommendations, the extent of response before surgery in patients who receive neoadjuvant therapy, and patient preferences. This customized approach to treatment requires a concerted, multidisciplinary effort shared among patients and radiology, pathology, genetics, and surgical, medical and radiation oncology providers to minimize adverse effects and preserve quality of life as much as possible [48].

The search for predictive biomarkers useful to draw further distinctions among BC subtypes is an active field of research that includes genomic, proteomic and/or machine learning approaches. In recent years, epigenetic biomarkers have gained growing attention, especially micro-RNAs (miRNA or miR) that have been predicted (and in some cases, validated) as very promising BC markers [49,50,51] for early detection as well [52,53,54]; notably, some studies also concentrated on circulating miR, opening the way towards a minimally invasive diagnostic approach [55,56,57,58].

## 3. Epigenetics of BC and the Role of miR-125

### 3.1. microRNA Nomenclature

MicroRNAs are short (20–25 nucleotides), single-stranded, non-coding RNA molecules whose main function is gene expression control, mainly silencing. They exert this downregulation by binding the 3′ end of target mRNA(s) through sequence homology and promoting either their degradation or impairing their translation [59]. Over 2500 miR have been estimated to be encoded in the human genome, regulating over 60% of human genes [60]. In addition, thanks to imperfect sequence pairing, they can also bind multiple targets, thus amplifying their intracellular action.

Beyond the number identifier (ID, usually higher for miR described chronologically later), additional nomenclature rules are established to identify unequivocally each miR [61,62]. miRs with almost identical sequence are identified by a progressive lowercase letter after the identification number (miR-XXXa, miR-XXXb, etc.), while miRs with identical sequence but mapping to different genomic locations are indicated by a progressive number separated from the ID number by a dash (e.g., miR-XXX-1, miR-XXX-2, etc.). To further distinguish molecules of different species, an additional three-letter code and a dash may be added at the beginning of the miR name (i.e., hsa-miR-XXX indicates a human–*Homo sapiens*–miR). Finally, the mature, single-stranded miR can be obtained either from the 5′ end or the 3′ end of its double-stranded miR precursor (pre-miR). Notably, sometimes both strands can—separately—be used for mRNA regulation, resulting in 5p and 3p miR forms if both are present and functional in the cell and the two miR are roughly equivalent in their intracellular amount; instead, if both are present but one is significantly more abundant than the other, then the rarer one has an asterisk at the end of its name (e.g., miR-XXX-5p*).

### 3.2. Role of miR in BC

Increasing evidence shows that miRs represent a central hub of gene expression control in human carcinogenesis, and, from this perspective, BC is not an exception [63,64].

Significantly, miR-21 has been shown to be responsible for the development of multidrug resistance [65], and it modulates the resistance of BC cells to doxorubicin by targeting *PTEN* [66]. In addition, miR-21 also plays a central role in BC proliferation and metastasis by targeting *LZTFL1* [67]. Additional miR-21 targets involved in cell proliferation, metastasis, epithelial-to-mesenchymal transition (EMT), and apoptosis in BC include *IGFBP3*, *TPM1*, *PCD4*, and *TGF-beta1* [68].

Another player in BC etiopathogenesis is miR-106a, which promotes cancer progression through the downregulation of *RAF-1* [69], *P53*, *BAX*, and *RUNX3* and the upregulation of *Bcl-2* and *ABCG2*; it also confers cisplatin resistance upon its upregulation [70,71].

Upregulation of miR-155 causes telomere fragility through its action on *TRF1*, a component of the shelterin complex [72]. Interestingly, this miR, together with miR-10b, miR-34a and miR-141, is also a possible candidate for building a panel of circulating miR useful for non-invasive detection of this tumor [73].

Conversely, downregulated miR-141 has been reported to be a typical feature of BC, where its target is *ANP32E* [74], which, in turn, induces tumorigenesis in triple-negative BC (TNBC) cells by upregulating *E2F1* [75]. Instead, high miR-141-3p expression is typical of grade III BC compared to grade II and, together with miR-181b1-5p and miR-23b-3p, it is a useful marker not only to discriminate between malignant and benign breast tissues but might also help in distinguishing TNBC from other molecular subtypes of BC [76].

The let-7 family of miR are tumor suppressors in several cancers, including BC, and their members can also be detected as circulating biomarkers [77]. Let-7 action involves the control of *ERCC6* expression [78], and its overexpression could inhibit BC cell proliferation.

Another circulating marker of BC is miR-335 [79], which exerts its effects by simultaneously regulating the known *BRCA1* activators *ERα*, *IGF1R*, *SP1* and the repressor *ID4*, including a feedback regulation of miR-335 expression by estrogens [80]. Its overexpression causes decreased cell viability and increased apoptosis, while other findings show it to negatively regulate the HGF/c-Met pathway, thus affecting cell scattering, migration, and invasion [81].

Another downregulated miR in BC is miR-126 [82]. Its targets include *VEGFA* and *PIK3R2* [83]. It also reduces trastuzumab resistance by targeting *PIK3R2* and regulating the AKT/mTOR signaling pathway [84] and controls cell invasion by targeting *ADAM9* [85].

Tumor suppressor miR-199a/b-3p inhibits migration and invasion of BC cells by downregulating the PAK4/MEK/ERK signaling pathway [86]. Overexpression of miR-199a-3p targets the c-Met and mTOR pathways, increases doxorubicin sensitivity and causes G1 phase arrest, thus reducing cell invasion and promoting doxorubicin-induced apoptosis [87]. This miR also confers resistance to cisplatin treatment by downregulating *TFAM* [88] and, at the same time, promotes BC development and metastasis under hypoxic conditions by controlling the regulatory axis consisting of HIF-1, SNHG1, and TFAM [89]. The same study mentioned above [87] also shows that in TNBC patients, additional circulating miR (i.e., miR-19a/b-3p, miR-25-3p, miR-22-3p, miR-210-3p and miR-93-5p) are deregulated as well and control several molecular pathways involved in drug resistance, making them amenable to be used as BC biomarkers, together with let-7a-5p, miR-100-5p and miR-101-3p, identified in another study [90].

Tumor suppressor miR-101 is downregulated as well in BC, and its targets include *POMP*, *Stmn1*, *DNMT3A*, *EYA1*, *VHL*, *SOX2*, *Jak2* and *MCL-1* (reviewed in [91]). For this reason, it plays a major role in the control of several cancer-related cellular processes, such as proliferation, apoptosis, angiogenesis, drug resistance, invasion, and metastasis. Overexpression of miR-101-3p can inhibit the migration of BC cells into the brain endothelium, a frequent and late event in BC patients, by inducing COX-2/MMP1 signaling, which can degrade the inter-endothelial junctions (claudin-5 and VE-cadherin) [92]. Jiang and collaborators showed that the suppression of the oncogene *EZH2* in BC by miR-101-3p is potentiated in the presence of syn-cal14.1a, a synthetic peptide derived from *Californiconus californicus* (a sea snail), thus inhibiting cell migration, invasion, and proliferation [93]. Additional data come from the work of Toda and collaborators, who performed an RNA-sequence-based microRNA expression signature in BC and identified other dysregulated miRs in BC (e.g., miR-99a-5p/-3p, miR-101-5p/-3p, miR-126-5p/-3p, miR-143-5p/-3p, and miR-144-5p/-3p) and found that miR-101-5p controls the expression of seven putative oncogenes (i.e., *HMGB3*, *ESRP1*, *GINS1*, *TPD52*, *SRPK1*, *VANGL1* and *MAGOHB*) [94].

Finally, miR-9 is known to exert critical functions in the initiation and progression of BC. Its upregulation—together with that of miR-221/222, miR-373 and miR-10b—is linked to highly malignant invasive EMT and cancer stem cell production [95]. Conversely, its downregulation can lead to improved overall survival, smaller tumors, earlier stages, and ER-positive cancers due to the enrichment of estrogen response genes [96]. Gwak and collaborators showed that miR-9 is highly expressed in HER2+ and TNBC subtypes compared with luminal subtypes, tumors with a high tumor stage or histologic grade, and tumors displaying the CD44+/CD24− phenotype, vimentin expression, and E-cadherin loss [97]. Interestingly, Shen and collaborators showed miR-9-5p, together with miR-195-5p and miR-203a-3p, to be a part of the extracellular vesicle (EV)-encapsulated miR (enabling cancer cell–cell communication in tumor pathogenesis and response to therapies) excreted upon docetaxel treatment [98]. As for its targets, genes identified so far include *FOXO1* [99], *STARD13* [100], *LIFR* [101], *elf5A2* [102], *HMGA2*, *EGR1*, and *IGFBP3* [68] and *PDGFRbeta* [103]. In turn, its expression is activated by MYC and MYCN [104,105].

A summary of the miR involved in BC and their function is summarized in Table 3.

All together, these data point to the involvement of several miRs in BC formation, development, metastasis, and drug resistance, showing that at the molecular level it is crucial to identify which pathways are altered and why, for example, the same gene may be deregulated because of the alteration of diverse miR. Knowing which miR is altered may greatly affect therapeutic approaches, especially in terms of avoiding cross-effects due to off-target actions. Thus, there is a necessity to identify (hopefully, all) the players in BC pathogenesis in a patient-specific way. In this perspective, the miR-125 family of miR has gained increasing relevance and attention in BC research, thanks to the numerous publications released over the last few years. In light of the numerous targets of these miRs and the multitude of pathways potentially altered inside the cell upon their dysregulation, in the next few years, miR-125 is likely to become central to understanding BC biology.

### 3.3. The miR-125 Family: Molecular Organization and Roles in Human Pathology

**Figure 1:**
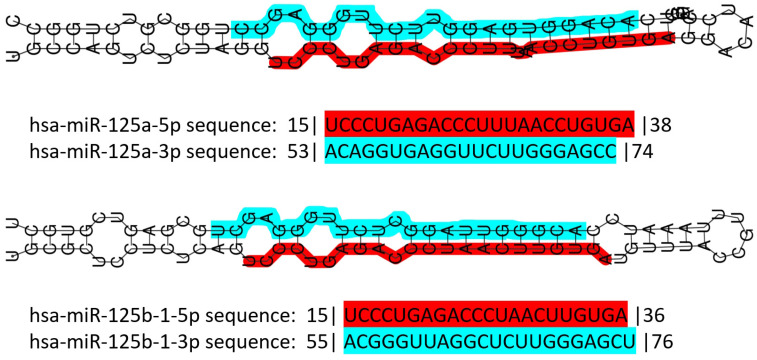
Schematic representation of human miR-125 illustrating the structure of the double-stranded pre-miR of both miR-125a and miR-125b, and their sequence. Color codes: red highlight for the 5p form, blue highlight for the 3p form. Numbers before and after the sequences indicate the number of nucleotides trimmed away from the mature miR. Data retrieved and partially modified from miRTarBase v9 update 2022 [112,113].

miR-125 is a highly conserved family of microRNAs whose members have also been found in nematodes (named lin-4 in 1993, the first miR described ever) [106]. The miR-125 family in *H. sapiens* includes three members, namely miR-125a, miR-125b-1 and miR-125b-2. The *MIR125A* gene maps to chromosome 19q13.41 [107], and miR-125a is part of a transcribed cluster of miR, together with miR-99b and let-7e [108]. The *MIR125B1* gene maps to chromosome 11q24.1, and in this locus, it is part of a cluster including the *LET7A2* and *MIR100* genes [108,109]. These miRs are inside the third intron of the *MIR100HG* gene [110]. Finally, the *MIR125B2* gene maps to chromosome 21q21.1, where it is included in a cluster together with the *MIR99A* and *LET7C* genes [108,109], inside the sixth intron of the *MIR99AHG* gene [110]. miR-125a and miR-125b differ only by a central diuridine insertion and a U-to-C change in miR-125a [111]. All members of the family show both 5p and 3p forms (Figure 1).

**Figure 2:**
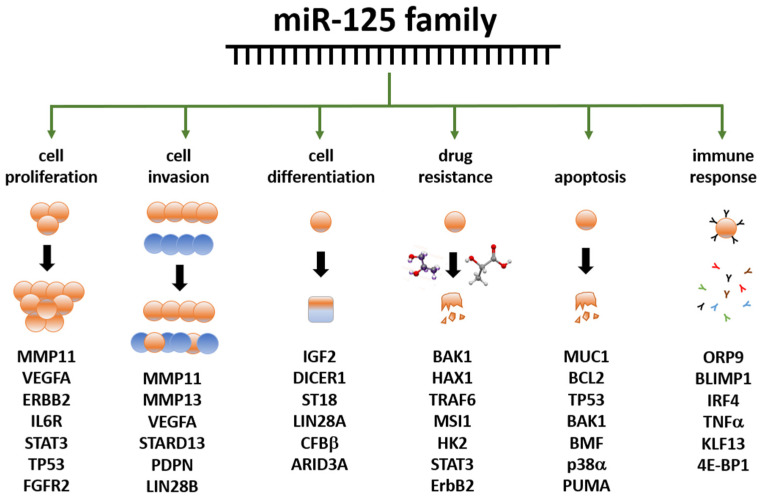
Summary of the main functions exerted by the miR-125 family members in human biology. Examples of miR-125 target genes are reported below each function. Data partly retrieved from [114,115] The listed genes at the bottom are involved in the control of the cellular functions below the green arrows; they are controlled, either directly or indirectly, by miR-125 family members, either by up- or down-regulation. Further details have been laid out in the article’s body.

The miR-125 family is involved in several cell metabolic pathways controlling differentiation, proliferation, apoptosis, metastasis formation, drug resistance and immune system function because of the targeting of mRNAs related to these cellular processes [114] (Figure 2). miR-125 molecules have a complex behavior inside the cell, which mirrors their expression pattern in different tissues/cell types [112,113], their ample variety of targets [112,113], the intracellular role of their targets, and the way miR and mRNAs are either up- or down-regulated upon expression.

The role of miR-125 family members has been extensively demonstrated in the muscle. It interacts with insulin-like growth factor II (*IGF-II*) to regulate myoblast differentiation in vitro and muscle regeneration in vivo [116], and with *TRAF6* to prevent atrophy [117]. It is also involved in the proliferation and migration of vascular smooth muscle cells induced by platelet-derived growth factor BB [118]. In cardiac muscles, miR-125 participates in the development of the heart in embryonic mammals (reviewed in [119]); it regulates muscle-enriched transcription factors in cardiac and skeletal myocytes [120]; it can modulate cardiac progenitor cell proliferation and migration potential [121]; and it regulates cardiomyocytes proliferation and apoptosis under oxidative stress conditions [122]. Cardiac-specific miR-125b deficiency has recently been shown to induce perinatal death and cardiac hypertrophy [123].

miR-125 is one of the most abundant microRNAs in the central nervous system (CNS) in both mice and men [124]. In humans, miR-125b promotes neuronal differentiation in human cells by repressing at least ten target mRNAs involved in those pathways [125,126]. It also regulates dendritic spine morphology and synaptic maturation [127], it is implicated in synaptic plasticity [128], promotes astrogliogenesis, and is involved in astrogliosis and glial cell proliferation [129]. Its deregulation has also been linked to CNS tumor formation and growth, such as pediatric low-grade glioma [130]; it regulates cell growth arrest and apoptosis of human neuroblastoma- and medulloblastoma-derived cell lines [131,132]; it inhibits cell apoptosis through p53 and p38MAPK-independent pathways in glioblastoma cells [133]; and, in glioma, it targets *BMF* [134].

In the immune system, miR-125 regulates hematopoiesis, inflammation, and immune cell function. miR-125a controls stem cell homeostasis during hematopoiesis [135,136,137,138] and plays a role in immune cell identity [138]. miR-125-5p targeting IL-6R regulates macrophage inflammatory response and intestinal epithelial cell apoptosis in ulcerative colitis through the JAK1/STAT3 and NF-κB pathways [139]. miR-125b-1-3p is expressed in hMSCs-Ad exosomes and can promote T lymphocyte apoptosis and alleviate atherosclerosis (AS) by down-regulating *BCL11B* expression, thus providing potential molecular targets for the clinical treatment of AS [140].

All together, these data emphasize the multiple roles of miR-125 family members in cell proliferation and differentiation in numerous body locations.

### 3.4. miR-125 and Cancer

Studying the role of miR-125 in cancer is an important research area; beyond the above-mentioned tumors of the CNS, this noncoding RNA is indeed deregulated in several other tumors [141]. Hematological cancers are the best-characterized malignancies in which miR-125 role is well established; due to the rather conclusive amount of findings available, we redirect the reader to specific and comprehensive reviews [142,143]. Additional organs affected by miR-125-related cancers include the ovary, bladder, liver, skin, bone, lung, pancreas, prostate, thyroid, stomach, colon and kidney. A summary of these cancers, known targets and related bibliographic references are reported in Table 4. A detailed description of the role of miR-125 family members in BC is reported in the next section.

### 3.5. Role of miR-125 in BC

A relatively small amount of research is currently available on the role of miR-125 in BC. The reports showing its altered expression in these malignancies started to be published more than 20 years ago, and the research is still running in search of an affordable diagnostic panel based on this noncoding RNA [182,183,184,185,186,187]. Among the targets first identified, it is worth mentioning *ERBB2* and *ERBB3* [188] mRNA. In 2011, Zhang and colleagues demonstrated the action of miR-125b on the regulation of the *ETS1* proto-oncogene in human invasive BC [189]. Rajabi et al. found that miR-125b, downregulated in BC, can reduce the expression of MUC1 (an oncoprotein), whose silencing causes DNA damage-induced apoptosis in cancer cells [190]. Tang and collaborators studied the effects of miR-125 deregulation on metastasis formation, finding that miR-125b induces metastasis by targeting *STARD13* mRNA in MCF-7 and MDA-MB-231 BC cells [191], in contrast with the tumor suppressive action described before. Using the same BC cell lines, Metheetrairut and collaborators showed that forced expression of miR-125b results in radiosensitivity, as seen by reduced clonogenic survival, enhanced apoptotic activity and enhanced senescence post-ionizing radiation treatment. Moreover, re-expression of c-JUN in MDA-MB-231 cells promoted radioresistance and abrogated miR-125-mediated radiosensitization, suggesting that overexpression of miR-125b causes sensibilization to γ-irradiation and indicating this miR as a possible target for adjuvant therapy [192]. In contrast, Wang et al. found an association between miR-125b expression and chemoresistance [193], again indicating an oncogenic role for this miR. In line with these last results, Zhou and collaborators found that miR-125b confers the resistance of BC cells to paclitaxel through suppression of pro-apoptotic *Bcl-2* antagonist killer 1 (*Bak1*) expression [194]. He and collaborators studied the expression of miR-125a-5p/3p and miR-125b in 143 pairs of BC and normal adjacent tissues, finding that miR-125a-5p and miR-125b were significantly down-regulated in BC tissue samples and that the expression level of miR-125a-5p was significantly higher in younger patients (<35 years) than in older ones, and a gradual reduction in miR-125a-5p expression was observed in BC tissue samples correlated to increasing age [195]. Recently, a paper showed the oncosuppressor role of miR-125b via the inhibition of proliferation, migration, and invasion of BC cells through targeting MMP11 protein expression [196]. A summary of the data reported above is illustrated in Table 5.

### 3.6. Further Mining miR-125 Function in BC: Competing Endogenous RNA Networks (ceRNET)

A fundamental way to control gene expression through miRs has been elucidated in recent years, consisting of the so-called ceRNET. In fact, miRs have been shown to act as controllers of target mRNAs by altering their half-life or translation. However, they are also controlled, in many cases, by other long non-coding RNAs (lncRNA) or even other mRNA, which “sponge” miR through sequence homology, avoiding their interaction with mRNA targets [197]. In other words, lncRNA and mRNA compete for binding miR; these two molecules form a competing endogenous RNA (ceRNA) couple. If the lncRNA efficiently sponges the miR, then miR inhibitory action is not accomplished, and the target mRNA is regularly translated. In this case, the lncRNA, inhibitor of an inhibitor, has a function resembling that of an enhancer of gene expression. Hence, if the mRNA encodes an oncoprotein, the lncRNA has an oncogenic effect, while the miR has an oncosuppressive role. The same, with opposite effects, occurs in the case of the mRNA coding an oncosuppressor. The three molecules, taken together, form what is currently known as a regulatory axis, and the sum of many axes creates the ceRNET. Here, lncRNA and mRNA constitute the nodes of the network, while miR represent their connections. A growing number of research works have been published in recent years outlining the increasing structure and complexity of the ceRNET in BC (see [198] and references therein), including the action of pseudogenes in this phenomenon. In fact, Welch and collaborators found that 309 pseudogenes exhibit significant differential expression among BC subtypes, and their expression pattern allows recognizing tumor samples from normal samples and discriminating the basal subtype from the luminal and Her2 subtypes; of them, 177 transcribed pseudogenes possess binding sites for co-expressed miRs that are also predicted to target their parent genes [199]. Recently, in a work by Zhu and collaborators, the authors took advantage of the data available in the exoRbase database and derived it from the exosomes of human BC samples [200]. Their study allowed for the identification of a ceRNA network including 19 mRNA nodes, 2 lncRNA nodes, 8 circular RNA nodes, and 41 miR connections. KEGG enrichment analysis showed that differentially expressed mRNA in the regulatory network is mainly enriched in the p53 signaling pathway.

**Figure 3:**
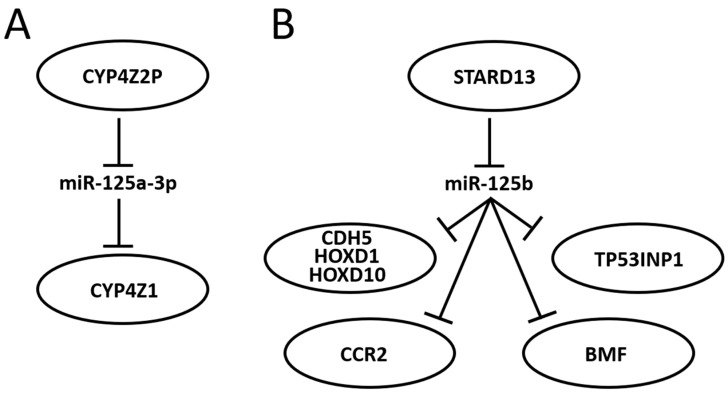
Schematic representation of two miR-125-centered ceRNET in BC. (**A**): a simple ceRNET having only one axis, where a pseudogene (*CYP4Z2P*) mRNA inhibits miR-125a-3p action on the target (*CYP4Z1*) mRNA by sponging it, thus enhancing *CYP4Z1* expression. (**B**): a more complex ceRNET in which the interaction between miR-125b and *STARD13* mRNA controls the expression of multiple target mRNA. See text for references and further explanations.

Research centered around portions of a miR-125-centered ceRNET has been expanding steadily. The miR-125 interactions with the mRNA described in the previous section are therefore likely to become axes of the growing BC ceRNET as well, as soon as the appropriate lncRNA is identified in the pathway. However, some axes have already been described, and some of them, being interconnected, can be used to build a basic version of this network (Figure 3).

In 2004, Rieger and collaborators discovered a new human cytochrome P450 (CYP), termed CYP4Z1, which is specifically expressed in mammary gland and breast carcinoma [201]. They also found a transcribed pseudogene, named *CYP4Z2P*, that codes for a truncated CYP protein (340 amino acids vs. 505) with 96% identity to CYP4Z1. Both CYPs are highly expressed in BC, although the expression level of *CYP4Z2P* is approximately 20 times lower than that of *CYP4Z1* in mammary tissues and barely expressed elsewhere. Later, it was shown that increased expression of *CYP4Z1* promotes tumor angiogenesis and growth in human BC [202] and that *CYP4Z2P* 3′-UTR is involved in promoting BC angiogenesis through the VEGF/VEGFR2 pathway [203]. In 2015, Zheng et al. showed that the action of *CYP4Z2P* 3′-UTR is sponging several miRs, including miR-125a-3p, and that this pseudogene acts as a ceRNA with respect to *CYP4Z1* mRNA, enhancing its expression levels [204]. They also showed that tumor angiogenesis is promoted by overexpression of the CYP4Z2P and CYP4Z1-3′UTRs, which significantly increased the activation of the ERK1/2 and PI3K/Akt pathways through the induction of their phosphorylation. The same group also showed a number of interactions later: (i) deregulation of these ceRNA also confers tamoxifen resistance in BC through the enhancement of the transcriptional activity of ERα via its phosphorylation dependent on cyclin-dependent kinase 3 (CDK3) [205]; (ii) downregulation of *CYP4Z1* or *CYP4Z2P* through 3′-UTR binding promotes cell apoptosis, mirroring the functions and modulating the expression of human telomerase reverse transcriptase (hTERT) [206]; (iii) transcriptional factor six2 activates these CYPs ceRNET by directly binding to their promoters, thus activating the downstream PI3K/Akt and ERK1/2 pathways and consequently being involved not only in chemoresistance but also regulating the stemness of BC cells [207].

STARD13 (StAR-related lipid transfer domain protein 13, also known as deleted in liver cancer 2 protein (DLC-2)) is a Rho GTPase-activating protein (Rho GAP) that selectively activates RhoA and CDC42 and suppresses cell growth by inhibiting actin stress fiber assembly in hepatocellular carcinoma (HCC) [208]; this protein is ubiquitously expressed in normal tissues and downregulated in HCC. In mice, STARD13 promotes angiogenesis through the actions of RhoA [209]. Its role is well established in BC as well, where it acts as a tumor suppressor gene [210], regulates cell motility and invasion [211], endothelial differentiation [103], metastasis formation [212,213], cell migration [214], and apoptosis [215]. It has also been shown that STARD13 exerts its function in BC through its participation in many ceRNETs, such as the one involving a positive TGF-β/miR-9 regulatory loop mediated by the STARD13/YAP axis [216], the one involving hsa-miR-21-3p [217], or even the more complex network that involves five different miRs and that controls YAP/TAZ nuclear accumulation and transcriptional activity via modulation of Hippo and Rho-GTPase/F-actin signaling pathways [218]. A direct link between miR-125 and *STARD13* expression has been described, too. Li and coworkers showed that *CDH5*, *HOXD1*, and *HOXD10* encode putative *STARD13* ceRNA and display concordant patterns with *STARD13* in different metastatic potential BC cell lines and tissues; in addition, they also show that the 3′ UTR of *STARD13* mRNA can bind miR-125b (and also miR-9 and miR-10b), indicating that this mRNA may participate in multiple pathways simultaneously [219], thus confirming their previous study about this interaction [191] and showing that the transcripts of the tumor suppressor genes *CDH5*, *HOXD1*, and *HOXD10* inhibit BC metastasis in vitro and in vivo by competing with *STARD13* mRNA for these three miR. Interestingly, *CDH5*, *HOXD1* and *HOXD10*, along with *STARD13*, are BC players also in a different ceRNET, competing for a different set of miRs, indicating that *STARD13′s* role in BC is very complex. In 2017, Hu et al. discovered another ceRNET axis in which *STARD13* and miR-125b control *CCR2* (*cysteine–cysteine chemokine receptor 2*) expression levels [212]. In this case, the authors found that the *CCR2* 3′ UTR harbors three miR-125 binding sites that both inhibit MDA-MB-231 and MCF-7 cell metastasis by repressing epithelial-mesenchymal transition (EMT) in vitro and suppress BC metastasis in vivo through competition with *STARD13* in a miR-125b-dependent and protein-coding-independent manner. Another component of the same ceRNET is *TP53INP1* (tumor protein p53-inducible nuclear protein 1). TP53INP1 is an antiproliferative and proapoptotic protein involved in cell stress response that acts as a dual regulator of transcription and autophagy and is modulated by p53 in response to stress; it also interacts with kinases HIPK2 and PKCδ, which phosphorylate p53, creating a positive feedback loop between p53 and TP53INP1 [220]. TP53INP1 is also involved in SPARC (secreted protein acidic and rich in cysteine)-mediated-promotive effects on cancer cell migration and metastasis [221]. In 2018, Zheng et al. found a ceRNA interaction between *STARD13* and *TP53INP1* mediated by competitively binding to miR-125b in BC [222]. In this case, *STARD13* promotes upregulation of *TP53INP1*, causing the inhibition of BC cell metastasis through competitive binding to miR-125b thanks to the inhibition of *SPARC* gene expression. Later, Guo and co-workers also found a ceRNET axis in BC involving miR-125b, *STARD13* and *BMF* (*Bcl-2-*modifying factor) mRNA [215]. BMF is a member of the BCL2 protein family and controls apoptosis in several cell types [223]. The authors [215] found that miR-125b directly binds the 3′ UTR and thus downregulates *BMF* expression, and that *STARD13*, sponging miR-125b, upregulates *BMF* in BC both in vitro and in vivo. All together, these results suggest novel therapies for BC treatment and aid in selecting adequate drugs, depending on the molecular biology of the tumor, from a perspective aiming at the goal of personalized medicine. Indeed, a recent study showed that tanshinone IIA (an effective component extracted from *Salvia miltiorrhiza* that regulates the stemness of tumor cells) attenuates this phenotype in BC cells by downregulating miR-125b levels and upregulating its target gene *STARD13* expression, while miR-125b overexpression or *STARD13* knockdown impairs the inhibitory effects of tanshinone IIA on the stemness of BC cells [224].

## 4. Discussion

BC is a heterogeneous disease; thus, patients that are histologically diagnosed with the same cancer type might have different molecular characteristics, genetic mutations or tumor microenvironments that can deeply influence the prognosis or treatment response. Consequently, the challenge in personalized medicine is to distinguish these diverse molecular characteristics, separate patients accordingly, and treat them using a tailored approach that considers all these features. Personalized medicine might profoundly improve patient outcomes thanks to diagnostic tests capable of identifying specific biomarkers, thus enabling doctors to select the most effective treatment for each patient, reduce the risk of adverse reactions and increase the likelihood of a successful outcome.

To better understand the complexities and implications from the ethical, legal and social perspectives of personalized early detection and prevention of BC, it is necessary to rely on recommendations and evidence-based criteria issued by scientific and policy institutions, e.g., the European Collaborative on Personalized Early Detection and Prevention of Breast Cancer (ENVISION) in its 2020 consensus statement [225]. Such guidance is all the more essential, in fact, when highly innovative practices and techniques are applied, whose potential growth may outpace our current ethics and legal criteria [226,227]. As ENVISION points out, in fact, there is no denying that a great deal of progress has been made in evidence-based personalized interventions capable of maximizing the benefits and mitigating the downsides of currently available BC screening and prevention programs. Such progress has resulted in substantial research innovations for assessing an individual woman’s risk of developing BC and relies on key factors such as the implementation of risk stratification models in BC prevention studies, achieving an effective degree of benefit–harm balance of risk-stratified early detection approaches, and the evaluation of the acceptability and feasibility of programs aimed at prevention and screening. Such a degree of innovation needs to be transposed into health outcomes for all; to achieve that, it is of utmost importance to devise and put in place a systematic approach for the assessment of risk-based programs, to be implemented along with thorough counseling being provided to patients in a highly targeted and tailored fashion [228]. In light of such needs, the classification of patients in the most precise way, at the molecular level, should be prioritized in order to better understand the biological features of the tumor to be treated. In this context, miR-125 and its targets are emerging as promising biomarkers in BC classification.

It is noteworthy that, depending on the study, miR-125 has been described as having either oncogenic or oncosuppressive roles. However, this should not be surprising. We recall that, for their very nature, the action of any miR is strictly connected with that of its targets; thus, if its target is an oncogene, then miR-125 acts as an oncosuppressor, and vice versa (see Table 4 and Table 5). However, the situation is further complicated for at least three reasons: cell changes in space (i.e., different regions of the same tumoral mass, which influence the cell response according to its diverse neighborhood), time (i.e., how the biology of the tumor changes over time), and miR-125 regulation. Firstly, different cancers, and sometimes also different stages of the same cancer, or even different populations of the same tumoral mass, have different metabolic needs [229]. Therefore, in the presence of both the same miR and corresponding mRNA target, the effect on the tumoral cell metabolism may significantly vary, with different spatial effects of miR on the transcriptome. Second, the evolution of cancer during time is associated with alterations in the cancer cell proteome; new genes are activated, other genes are suppressed or lost (for example, through aneuploidy [230] or copy number variations [231,232]) and also as a response to the internal and external microenvironmental interactions [233,234,235]. This is obviously true for every single cancer subpopulation (the space variable described before). Third, we recall here the organization of the miR-125b genes: this miR is transcribed from two different loci in the genome, which are under two different promoters [108]. Consequently, it is quite straightforward to hypothesize that the two copies of miR-125b may not be fully interchangeable in their function despite their sequence identity, since they may be transcribed under different cellular conditions and, thus, bind different targets at the time of miR-125b expression. In addition, the two clusters where miR-125b is embedded vary in their contents; the transcription of the cluster as a single pri-miR using the same promoter suggests that these diverse miR need to act in concert, thus the two different clusters, upon expression, likely modify the host cell proteome in different ways. It is therefore possible to theorize that such inconsistencies in the role of miR-125 (oncogene vs. oncosuppressor) might just reflect differences in the cells analyzed or in the analytical protocols applied, rather than real contradictions. Moreover, additional variables might be taken into account to explain these inconsistencies; as mentioned earlier, the same cell in a different microenvironment could respond differently to both internal (e.g., mutations, nutrient shortages, oxidative stress) and external (cell–cell interactions, response to immune system attack) stimuli, thus further encouraging the molecular characterization of each patient is arguably becoming a priority. Indeed, some support for this explanation is available for BC cell lines used to verify miR-125 function. In fact, miR-125 has been shown to be expressed in MCF-7 spheroids but not in MDA-MB-231 spheroids; in addition, the unique cluster of miRs found in each cell type is reportedly associated with their chemoresistance properties and cancer progression, most likely influencing the maintenance of the spheroid-enriched cancer stem cell properties [236]. Similar differences in different BC cell lines have also been reported by Ahram and coworkers who compared MDA-MB-453, MCF-7 and T47D cells, finding that miR-125b is highly expressed in T47D cells and slightly downregulated in MDA-MB-453 cells, with all the predictable consequences related to their target fate [237]. All together, these data underline the importance and complexity of the expression of miR-125 family members in the etiopathogenesis of BC and the need to characterize this biomarker further and better in BC patients.

## 5. Conclusions

It is noteworthy that, at present, the role of miR-125 in BC is quite underestimated in clinical practice. A search on the website clinicaltrial.gov performed in November 2023, using BC and microRNA as keywords, retrieved only 21 hits; of them, only one (ID: NCT04778202) is aimed at studying ‘miR 125a-5p and miR 143-3p as non-invasive biomarkers in the diagnosis of BC and the relationship between miR expression and histopathological features as tumor stage, grade, molecular subtypes’; for this trial, however, recruiting has not started yet [238]. No clinical trial is presently planned to study miR-125 as a possible therapeutic agent to control gene regulation in selected patients with altered expression of known target genes, despite its growing importance (see Figure 2 and Figure 3, and Table 5). Yet, by its very nature, this molecule is remarkably challenging in its clinical use. The usual approach to silence or enhance the function of a dysregulated miR basically relies on two approaches: (i) to restore miR expression with tumor suppressing activity (gain of function) or (ii) to block miR with oncogenic activity inhibiting its function (loss of function) [239]. Such approaches, however, are not fully applicable to miR-125, especially if it is a direct target of the therapy. For the silencing, both strands (5p and 3p) have a biological function in the cell, so the risk of inhibiting one strand by upregulating the other is high. For the enhancement obtained, for example, through the ectopic expression of an artificial construct, the fact that miR-125 is co-transcribed with other miR complicates this approach because all co-expressed miR need to be characterized, quantified, and possibly re-regulated. For this reason, directly targeting the miR would be, in our view, very complicated. Instead, it would be easier to target the locus activity harboring miR-125 so that co-expressed miRs are synchronously regulated. Naturally, this requires a profound knowledge of the locus organization, including the presence of enhancers, silencers, other regulatory elements, and chromatin modifications, and the study on miR-125 is, unfortunately, not so advanced in this perspective. At the moment, however, miR-125 has a potentially great impact on clinical practice as a biomarker, either in biopsies or as a circulating molecule, and the technology is fully proficient to perform such kinds of analyses.

Personalized medicine undoubtedly constitutes a broad-ranging breakthrough with huge potential to change healthcare to its very core. At the same time, such a potential will likely bring about a sea-change in that the current sets of ethical, legal and policy-making standards that provide us guidance today may be outpaced and ultimately inadequate. Therefore, new criteria need to be devised if we are to rely on equitable, effective healthcare for all in the long term. These criteria need to be supported by scientific discoveries, and likely miR-125 will be highly relevant and meaningful over the next few years in BC diagnosis and treatment.

## Figures and Tables

**Table 1:** Main genes associated with BC formation and development. Data in columns 3–4 partly retrieved from [25,26,27,28]. Estimated risk refers to the probability to develop a BC in presence of a mutation in the corresponding gene. Abbreviations: TNBC—triple negative breast cancer; BC—breast cancer; n/a: data not available (e.g., low risk gene, insufficient data available, non-specific effect); refs—bibliographic references.

Gene	Function(s)	Estimated Risk	BC Type	Refs
BRCA1	DNA repair transcription regulation cell cycle regulation chromatin remodeling	55–65% by age 70	TNBC luminal B	[11,25,27,28]
BRCA2	DNA repair DNA replication transcription regulation cell cycle regulation mitophagy	~45% by age 70	TNBC luminal B	[11,25,28]
PALB2	DNA repair	All women: RR 2.3, 95% CI 1.4–3.9 < 50 years: RR 3.0, 95% CI 1.4–5.5	n/a	[17,25,28]
PTEN	cell survival cell growth	85% lifetime	luminal A luminal B	[12,25,26,27,28]
TP53	cell cycle regulation	25% by age 74	all	[13,25,26,27,28]
CDH1	cell adhesion	39% lifetime	luminal A	[14,25,26,27,28]
STK11	cell cycle regulation	32% by age 60	n/a	[15,25,28]
CHEK2	DNA repair cell cycle regulation apoptosis	Female: RR 1.70, 95% CI 1.3–2.2 Male: RR 10.3, 95% CI 3.5–30.0	n/a	[16,25,26,27,28]
BRIP1	DNA repair	All women: RR 2.0, 95%	n/a	[23,25]
ATM	DNA repair	RR 2.37, 95% CI 1.5–3.8	n/a	[18,25,26,27,28]

**Table 2:** TNM (tumor-node-metastasis) staging for BC. A given BC can be identified by any combination of T-N-M parameters, based on patient’s clinical status.

Tumor		Node		Metastasis	
Tx	no primary tumor information	Nx	not assessable	Mx	not assessed
T0	no primary tumor evidence	N0	no clinically positive nodes	M0	no evidence
TIS	carcinoma in situ (primary sites)	N1	single, ipsilateral, size < 3 cm	M1	metastasis present at distance
T1	size < 2 cm	N2a	single, ipsilateral, size 3–6 cm		
T2	size 2 to 4 cm	N2b	multiple, ipsilateral, size < 6 cm		
T3	size > 4 cm	N3	massive/ipsilateral/bilateral/controlateral		
T4	size > 4 cm, pterygoid muscle, base of tongue or skin involved	N3a	ipsilateral node(s), one more than 6 cm		
		N3b	bilateral		
		N4	controlateral		

**Table 3:** List of miR playing a direct role in BC. Target genes are those for which the miR/mRNA interaction is direct (usually, at the mRNA 3′ UTR), thus indirect interactions (e.g., other proteins of the same metabolic axis) are not reported in this table; additional miR studied only as BC biomarkers are collectively reported in the bottom row; see text for additional explanations. Abbreviations: n/a—data not available.

miR Name	Target Gene(s)	Affected Cellular Functions	Refs
miR-21	PTEN	drug resistance	[65,66]
miR-21	LZTFL1	proliferation and metastasis	[67]
miR-21	IGFBP3 TPM1 PCD4 TGF-β1	proliferation, metastasis, epithelial-to-mesenchymal transition (EMT), apoptosis	[68]
miR-106a	RAF-1	invasion and proliferation	[69]
miR-106a	P53 BAX RUNX3 Bcl-2 ABCG2	proliferation, colony-forming capacity, migration, invasion, apoptosis, sensitivity to cisplatin	[70,71]
miR-155	TRF1	telomere fragility	[72]
miR-141	ANP32E	migration and invasion	[74]
let-7	ERCC6	proliferation, apoptosis	[78]
miR-335	ERα IGF1R SP1 ID4	proliferation, apoptosis	[80]
miR-335	c-Met	cell scattering, migration, and invasion	[81]
miR-126	VEGFA PIK3R2	angiogenesis, tumor genesis and growth	[83]
miR-126	PIK3R2	trastuzumab resistance	[84]
miR-199a/b-3p	PAK4	migration and invasion	[86]
miR-199a-3p	mTOR c-Met	cell cycle progression, doxorubicin sensitivity, apoptosis	[87]
miR-199a-3p	TFAM	resistance to cisplatin	[88]
miR-199a-3p	TFAM	angiogenesis and metastasis under hypoxia	[89]
miR-101	POMP Stmn1 DNMT3A EYA1 VHL SOX2 Jak2 MCL-1	proliferation, apoptosis, angiogenesis, drug resistance, invasion, metastasis	[91]
miR-101-3p	COX-2	migration, metastasis	[92]
miR-101-3p	EZH2	migration, invasion, proliferation	[93]
miR-101-5p	GINS1	DNA replication	[94]
miR-9	FOXO1	proliferation, migration, invasion	[99]
miR-9	STARD13	EMT, metastasis	[100]
miR-9	LIFR	metastasis	[101]
miR-9	elf5A2	resistance to doxorubicin	[102]
miR-9	HMGA2 EGR1 IGFBP3	proliferation, metastasis, EMT, apoptosis	[68]
miR-9	PDGFRβ	vasculogenesis	[103]
miR-200	PDGFRβ	vasculogenesis	[103]
let-7a-5p miR-9-5p miR-10b miR-21 miR-22-3p miR-23b-3p miR-25-3p miR-29 miR-34a miR-93-5p miR-99a-5p/-3p miR-100-5p miR-101-3p miR-101-5p miR-126-5p/-3p miR-141-3p miR-143-5p/-3p miR-144-5p/-3p miR-145 miR-155 mir-181b1-5p miR-195-5p miR-199a-5p miR-200a miR-203 miR-203a-3p miR-205 miR-210-3p miR-221/222 miR-373	n/a	biomarkers	[73,76,87,90,94,95,97,98]

**Table 4:** Summary of the affected organs and mRNA targets of miR-125 family members in human cancers. Data regarding BC is reported in Section 3.5. Abbreviations: CNS—central nervous system; refs—references; n/a—data not available in the cited reference(s). In the “notes” column, data refers to reported anomalies in miR-125 regulation, to its action on specific pathways, or to additional data that might explain its role in the specific cancer; in case nothing is relevant—beyond the identified target genes—we report “none.” Reported sources can be broadly divided into two classes: those investigating deregulated miR in cancer samples (for which target identification is usually absent) and those investigating miR-125 functional role(s), for which the main aim of the study is reported in the first three columns.

miR	Organ	Target(s)	Notes	Refs
125	CNS	n/a	deregulated, pediatric	[130]
125	CNS	n/a	deregulated	[131,132]
125	CNS	p53, p38MAPK	none	[133]
125	CNS	BMF	none	[134]
125a	ovary	n/a	EMT negative regulator	[144]
125b	ovary	BCL3	none	[145]
125b	ovary	n/a	serum biomarker	[146]
125b	bladder	E2F3	none	[147]
125b	bladder	n/a	urine biomarker	[148]
125-3p	bladder	n/a	hypoxia regulated	[149]
125	bladder	n/a	survival predictor	[150]
125a	liver	MMP11, VEGF	none	[151]
125b	liver	Mcl-1, IL6R	none	[152]
125b	liver	Lin28B2	none	[153]
125	liver	Pokemon	none	[154]
125	liver	TRAF6	none	[155]
125	liver	hexokinase II	none	[156]
125	liver	FOXM1	none	[157]
125	skin	NCAM	none	[158]
125	skin	c-Jun	none	[159]
125b	skin	MMP13	none	[160]
125b	skin	STAT3	none	[161]
125	skin	n/a	deregulated	[162]
125b	bone	STAT3	none	[163]
125	bone	ErbB2	none	[164]
125	bone	BAP1	none	[165]
125	lung	n/a	survival predictor	[166]
125	lung	EGFR	none	[167]
125	lung	HER2	trastuzumab resistance	[168]
125	lung	MMP13	none	[169]
125	pancreas	n/a	deregulated	[170,171]
125	pancreas	NEDD9	none	[172]
125	prostate	n/a	deregulated	[173,174,175]
125	prostate	BAK1	none	[176]
125	prostate	p53, PUMA	none	[177]
125b	thyroid	Foxp3	cisplatin sensitivity	[178]
125b	stomach	PPP1CA-Rb	none	[179]
125a-5p	colon	BCL2, BCL2L12, MCL1	none	[180]
125b	kidney	n/a	survival predictor	[181]

**Table 5:** Role of miR-125 in BC formation and development. In columns 2 and 4, reg. stands for regulation; an arrow pointing upwards means upregulation, while an arrow pointing downwards means downregulation; each arrow describes the miR/target regulation reported to its left. Note that in BC models, miR-125 members may be either up- or down-regulated, indicating either an oncogenic or oncosuppressive role for this molecule, in that context. Possible interpretations of these contradictory data are reported in the Discussion. In columns 3–4, n/a stands for ‘data not available’ or ‘not applicable’. In column 6, ‘blood samples’ means that circulating miR have been studied. In column 7, ref. stands for reference(s).

miR	Reg.	Target	Reg.	Cellular Function	Cell Line	Ref.
miR-125a miR-125b	↑ ↑	ERBB2 ERBB3	↓ ↓	migration invasion	SKBR3	[188]
miR-125b	↓	ETS1	↑	proliferation	BC samples	[189]
miR-125b	↓	MUC1	↑	apoptosis	BT-549 ZR-75-1	[190]
miR-125b	↓	STARD13	↑	metastasis	MCF-7 MDA-MB-231	[191]
miR-125	↓	n/a	n/a	radioresistance	MCF-7 MDA-MB-231	[192]
miR-125b	↑	n/a	n/a	chemoresistance proliferation apoptosis	blood samples	[193]
miR-125b	↑	BAK1	↓	chemoresistance apoptosis	MDA-435 MDA-436 MDA-231 MCF7 SKBR3	[194]
miR-125a-5p miR-125b	↓ ↓	n/a	n/a	age-dependent BC formation	BC samples	[195]
miR-125b	↓	MMP11	↑	proliferation migration invasion	T47D SKBR3	[196]

## Data Availability

Not applicable.
