# Correction: Piergentili et al. miR-125 in Breast Cancer Etiopathogenesis: An Emerging Role as a Biomarker in Differential Diagnosis, Regenerative Medicine, and the Challenges of Personalized Medicine. *Non-Coding RNA* 2024, *10*, 16

**DOI:** 10.3390/ncrna11040050

**Published:** 2025-06-25

**Authors:** Roberto Piergentili, Enrico Marinelli, Gaspare Cucinella, Alessandra Lopez, Gabriele Napoletano, Giuseppe Gullo, Simona Zaami

**Affiliations:** 1 Institute of Molecular Biology and Pathology, Italian National Research Council (CNR-IBPM), 00185 Rome, Italy; roberto.piergentili@cnr.it; 2 Department of Medico-Surgical Sciences and Biotechnologies, “Sapienza” University of Rome, 04100 Latina, Italy; enrico.marinelli@uniroma1.it; 3 Department of Obstetrics and Gynecology, Villa Sofia Cervello Hospital, University of Palermo, 90146 Palermo, Italy; gaspare.cucinella@unipa.it (G.C.); alessandralopez91@gmail.com (A.L.); gullogiuseppe@libero.it (G.G.); 4 Department of Anatomical, Histological, Forensic and Orthopedic Sciences, Section of Forensic Medicine, “Sapienza” University of Rome, 00161 Rome, Italy; gabriele.napoletano@uniroma1.it

There was an error in the original publication [1]. Reference number 164: “Yang, Y.; Chen, Y.; Liu, J.; Zhang, B.; Yang, L.; Xue, J.; Zhang, Z.; Qin, L.; Bian, R. MiR-125b-5p/STAT3 Axis Regulates Drug Resistance in Osteosarcoma Cells by Acting on ABC Transporters. *Stem Cells Int.* **2023**, *2023*, 9997676. https://doi.org/10.1155/2023/9997676.” was retracted prior to the publication of this article. The authors were unaware of the retraction at the time of submission to *ncRNA* and have confirmed that reference number 164 is not essential to their paper.

Reference [164] was removed from Table 4, Epigenetics of BC and the Role of miR-125, in the section *miR-125 and Cancer*. The corrected Table 4 is shown below: 

With this correction, the order of some references has been adjusted accordingly. The authors state that the scientific conclusions are unaffected. This correction was approved by the Academic Editor. The original publication has also been updated.

## Figures and Tables

**Table 4:** Summary of the affected organs and mRNA targets of miR-125 family members in human cancers. Data regarding BC is reported in Section 3.5. Abbreviations: CNS—central nervous system; refs—references; n/a—data not available in the cited reference(s). In the “notes” column, data refers to reported anomalies in miR-125 regulation, to its action on specific pathways, or to additional data that might explain its role in the specific cancer; in case nothing is relevant—beyond the identified target genes—we report “none.” Reported sources can be broadly divided into two classes: those investigating deregulated miR in cancer samples (for which target identification is usually absent) and those investigating miR-125 functional role(s), for which the main aim of the study is reported in the first three columns.

miR	Organ	Target(s)	Notes	Refs
125	CNS	n/a	deregulated, pediatric	[130]
125	CNS	n/a	deregulated	[131,132]
125	CNS	p53, p38MAPK	none	[133]
125	CNS	BMF	none	[134]
125a	ovary	n/a	EMT negative regulator	[144]
125b	ovary	BCL3	none	[145]
125b	ovary	n/a	serum biomarker	[146]
125b	bladder	E2F3	none	[147]
125b	bladder	n/a	urine biomarker	[148]
125-3p	bladder	n/a	hypoxia regulated	[149]
125	bladder	n/a	survival predictor	[150]
125a	liver	MMP11, VEGF	none	[151]
125b	liver	Mcl-1, IL6R	none	[152]
125b	liver	Lin28B2	none	[153]
125	liver	Pokemon	none	[154]
125	liver	TRAF6	none	[155]
125	liver	hexokinase II	none	[156]
125	liver	FOXM1	none	[157]
125	skin	NCAM	none	[158]
125	skin	c-Jun	none	[159]
125b	skin	MMP13	none	[160]
125b	skin	STAT3	none	[161]
125	skin	n/a	deregulated	[162]
125b	bone	STAT3	none	[163]
125	bone	ErbB2	none	[164]
125	bone	BAP1	none	[165]
125	lung	n/a	survival predictor	[166]
125	lung	EGFR	none	[167]
125	lung	HER2	trastuzumab resistance	[168]
125	lung	MMP13	none	[169]
125	pancreas	n/a	deregulated	[170,171]
125	pancreas	NEDD9	none	[172]
125	prostate	n/a	deregulated	[173–175]
125	prostate	BAK1	none	[176]
125	prostate	p53, PUMA	none	[177]
125b	thyroid	Foxp3	cisplatin sensitivity	[178]
125b	stomach	PPP1CA-Rb	none	[179]
125a-5p	colon	BCL2, BCL2L12, MCL1	none	[180]
125b	kidney	n/a	survival predictor	[181]
